# Tomato ATP-Binding Cassette Transporter SlABCB4 Is Involved in Auxin Transport in the Developing Fruit

**DOI:** 10.3390/plants7030065

**Published:** 2018-08-13

**Authors:** Peter Amoako Ofori, Markus Geisler, Martin di Donato, Hao Pengchao, Shungo Otagaki, Shogo Matsumoto, Katsuhiro Shiratake

**Affiliations:** 1 Graduate School of Bioagricultural Sciences, Nagoya University, Chikusa, Nagoya 464-8601, Japan; oforiamoakopeter@yahoo.com (P.A.O.); sotagaki@agr.nagoya-u.ac.jp (S.O.); shogo@agr.nagoya-u.ac.jp (S.M.); 2 Department of Biology, University of Fribourg, CH-1700 Fribourg, Switzerland; markus.geisler@unifr.ch (M.G.); martin.didonato@unifr.ch (M.d.D.); pengcho.hao@gmail.com (H.P.)

**Keywords:** ABC transporter, auxin, fruit development

## Abstract

Plant ATP binding cassette (ABC) transporters are membrane proteins that are important for transporting a wide range of compounds, including secondary metabolites and phytohormones. In Arabidopsis, some members of the ABCB subfamily of ABC transporter, also known as Multi-Drug Resistance proteins (MDRs), have been implicated in auxin transport. However, reports on the roles of the auxin-mediated ABCBs in fleshy fruit development are rare. Here, we present that SlABCB4, a member of the tomato ABCB subfamily, transports auxin in the developing fruit of tomato. Transient expression of SlABCB4-GFP fusion proteins in tobacco cells showed plasma membrane localization. The transport activity of SlABCB4, expressed in *Nicotiana benthamiana* protoplasts, revealed substrate specificity for indole-3-acetic acid export. Gene expression analysis of SlABCB4 revealed high expression levels at the early stages of fruit development. Therefore, SlABCB4 is considered to facilitate auxin distribution in tomato fruit, which is important for tomato fruit development.

## 1. Introduction

In plants, ATP binding cassette (ABC) transporters have important physiological functions, such as the detoxification processes of cells, cuticle formation, stomatal regulation, seed germination and resistance to pathogens [1]. They are also involved in the transport of various compounds, including heavy metals, antibiotics, phytohormones and secondary metabolites [2]. In Arabidopsis, some members of the ATP binding cassette subfamily B (ABCBs), also known as Multi-Drug Resistance proteins (MDRs) of ABC transporters, are characterized as auxin transporters [3]. While it is evident that auxin-related ABCBs may play an important role in fruit development, their contributions in fleshy fruit development are relatively unknown.

Generally, the role of auxin in fruit development extends from flower formation through to fruit ripening [4]. Auxin interacts with gibberellin and cytokinin to initiate the fruit set after fertilization [5,6]. Many studies have found parthenocarpic fruit set results from either increased or decreased auxin transduction [7,8]. For instance, up-regulation of auxin, through exogenous application to the ovaries, results in seedless fruit formation, without the occurrence of fertilization [9,10]. The auxin signal is important to promote gibberellin synthesis in the ovule required to stimulate fruit growth [11,12]. Auxin is also suggested to be involved in fruit expansion [13], although supporting information for this assertion is scarcely available [4]. 

In fleshy fruit, such as tomato, the seed is believed to be the de novo site for auxin biosynthesis [4]. This is because the endogenous auxin, indole-3-acetic acid (IAA), is reportedly synthesized in relatively higher concentrations in the seed compared with other fruit tissues [14,15]. Although a high auxin level in seed is a prerequisite for embryo and endosperm development, it has been suggested that the seed is the primary source of auxin for distribution in other fruit tissues, and this promotes cell division and enlargement concomitant with fruit growth [16,17]. In the tissues of developing fruit, auxin transporter proteins regulate auxin distribution. To regulate auxin activities, auxin must be translocated to target tissues from synthesizing sites [18]. In plants, distribution of auxin is done by auxin transporters, which include PIN-FORMED (PIN), PIN-LIKES (PILS), AUXIN1/LIKE-AUX1 (Aux1/LAX), and some members of ABCBs [19]. 

In Arabidopsis, the function of these auxin-related transporters is extensively studied [4]. However, most of the reported findings are related to vegetative growth and development [18,20,21,22]. Among the auxin-related ABCBs, AtABCB1 and AtABCB19 are intensively characterized. The gene expression of *AtABCB1* is targeted to the root and shoot tissues, whereas AtABCB19 is present in whole plants [2]. Functional analysis of AtABCB1 and AtABCB19 showed auxin transport activity in the hypocotyl and stem [2,23]. The roles of PIN [14,19] and Aux/LAX [14] have been characterized in fleshy fruits development [5], while reports on the roles of the auxin-mediated ABCBs in fleshy fruit development are rare. In tomato, the *PIN* and *LAX* genes display fruit developmental-specific expression patterns, suggesting the importance of auxin distribution by these auxin transporters in various fruit developmental processes [4,14]. For instance, down-regulation of the *SlPIN4* [24] gene in tomato resulted in the parthenocarpic fruit set. 

Tomato is an important horticultural crop and used as a model plant for fleshy fruit studies. In our recent studies, a genome-wide analysis of ABC transporters in tomato was performed. It highlighted the potential roles of ABC transporters in tomato fruit development. In a previous study, SlABCB4 was found to be a close homolog of the Arabidopsis auxin transporter, AtABCB19, and showed high gene expression in developing tomato [25]. Hence, SlABCB4 was selected for further studies to clarify its potential roles in tomato fruit development. In this study, we performed the functional characterization of SlABCB4 and suggested its importance in tomato fruit development. 

## 2. Materials and Methods 

### 2.1. Plant Materials

Tomato (Solanum lycopersicum) ‘MicroTom’ seeds were obtained from the National Bioresource Project (NBRP)-Tomato (http://tomato.nbrp.jp/indexEn.html) with an accession number, TOMJPF00001. Plants were grown in a growth chamber (Biotron LPH-350S, NK Systems, Minato-ku, Tokyo, Japan) which was set to a constant 25 °C, 60% relative humidity and 16 h light/8 h dark photoperiod. Plants were watered with tap water twice a week. A half concentration of Otsuka fertilizer was also applied once per week. 

Tomato tissues were sampled according to the method described by Reuscher et al. [26]. Vegetative tissues, including stems, roots, young and matured leaves, were sampled from 6-week-old plants. Fully expanded and unexpanded leaves were sampled as mature and young leaves, respectively. Reproductive tissues consisted of fully opened flowers and developing fruit samples at 3, 7, 14, 21 and 28 days after pollination (DAP), and breaker and red stages were sampled and stored in liquid nitrogen.

### 2.2. Phylogenetic Analysis

Protein sequences of the tomato’s full-size ABCB were retrieved from Sol Genomics Network (https://solgenomics.net/). Members of the Arabidopsis full-size ABCB subfamily were obtained from the phytozome database (https://phytozome.jgi.doe.gov/pz/portal.html). The protein sequences were aligned using the CLUSTALW program (http://www.genome.jp/tools-bin/clustalw) [27] and a phylogenetic tree was generated using the neighbor joining method of the MEGA06 software [28].

### 2.3. RNA Extraction and Reverse Transcription-Quantitative (RT-qPCR)

To perform the RT-qPCR analysis, total RNA was extracted from collected samples using Trizol reagent (Invitrogen, Carlsbad, CA, USA) for vegetative tissues and the hot borate method [29] for reproductive tissues. The cDNA was synthesized using the PrimeScript RT reagent kit (Takara Bio Inc., Kusatsu, Japan). RT-qPCR was performed using the SYBR Premix ExTaq II (Takara Bio Inc., Kusatsu, Japan) and the Thermal Cycler Dice Real Time (Takara Bio Inc., Kusatsu, Japan). Gene-specific primers used are shown in Supplementary Table S1. Ubiquitin (SlUBQ, Solyc01g056940) was used as an internal control [14]. For each sample, the RT-qPCR analysis was performed on three biological replicates and three technical repeats. Statistical analysis was performed using the Microsoft Excel Statistics 2013 for Windows.

### 2.4. Subcloning of cDNA of SlABCB4 into Plant Expression Vectors

A full length cDNA clone of SlABCB4 was obtained from the National Bioresource Project (NBRP)-Tomato (http://tomato.nbrp.jp/indexEn.html) with clone ID number, LEFL2031I14. The KOD, plus DNA polymerase (Toyobo, Osaka, Japan), and pENTR D-TOPO Cloning Kit (Invitrogen, Carlsbad, CA, USA) were used. A full length cDNA of SlABCB4 was cloned into the pENTR D-TOPO entry vector (Invitrogen, Carlsbad, CA, USA) using the In-Fusion cloning system (Takara Bio Inc., Kusatsu, Japan), following the method described by Park et al. [30]. In brief, amplification of the entry clone and linearization of the entry vector was done by using the in-fusion primers, which were generated using the In-Fusion cloning online tools (Takara Bio Inc., Kusatsu, Japan) (Supplementary Table S1). The Cauliflower mosaic virus 35S promoter driven expression constructs with no tag, C- and N-terminal GFP tags; pGWB2-SlABCB4, pGWB5-SlABCB4-GFP and pGWB6-GFP-SlABCB4 [31], respectively, were generated using the Gateway LR reaction (Invitrogen). 

### 2.5. Subcellular Localization

pGWB5-SlABCB4-GFP and pGWB6-GFP-SlABCB4 were transiently expressed in leaf tissue of *Nicotiana benthamiana* by Agrobacterium-mediated transfection [32]. The plasma membrane protein marker, 138R-Wave (UBQ10: mCherry-PIP1; 4) [33], was co-transfected with the vectors to express SlABCB4-GFP fusion proteins. In addition, the plasma membrane of the *N. benthamiana* epidermal leaf cells was stained with lipophilic dye, FM4-64. Imaging of the epidermal cell was done by using a confocal laser scanning microscope (TCS SP5, Leica, Wetzlar, Germany).

### 2.6. Auxin Transport Assay

pGWB2-SlABCB4, pGWB5-SlABCB4-GFP and pGWB6-GFP-SlABCB4 were transiently expressed in leaf tissue of *N. benthamiana* by Agrobacterium-mediated transfection [32]. Preparation of *N. benthamiana* mesophyll protoplasts and measurement of indole-3-acetic acid (IAA) and benzoic acid (BA) exports from protoplasts were performed according to the procedures of Geisler et al. [34]. In brief, the isolated protoplasts were preloaded by incubation with 1 µm/mL each of [^3^H]IAA with a specific activity of 9.3 × 10^11^ Bq mmol^−1^ (American Radiolabelled Chemicals) and [^14^C]BA with a specific activity of 2.0 × 10^9^ Bq mmol^−1^ (American Radiolabelled Chemicals) on ice for 10 min, followed by Percoll gradient centrifugation to isolate the loaded protoplasts from external radioactivity. To begin the export assay, isolated protoplasts were incubated at 25 °C and stopped by silicon oil centrifugation. The radioactivity in the protoplasts was counted by scintillation counting. Exported radioactivity was calculated as the relative efflux prior to 25 °C incubation. The experiment was repeated at least four times with three replicates each. Mean values were compared using the least significant difference (LSD) test at *p* < 0.05 level, using the Microsoft Excel Statistics 2013 for Windows.

## 3. Results and Discussion

**Figure 1:**
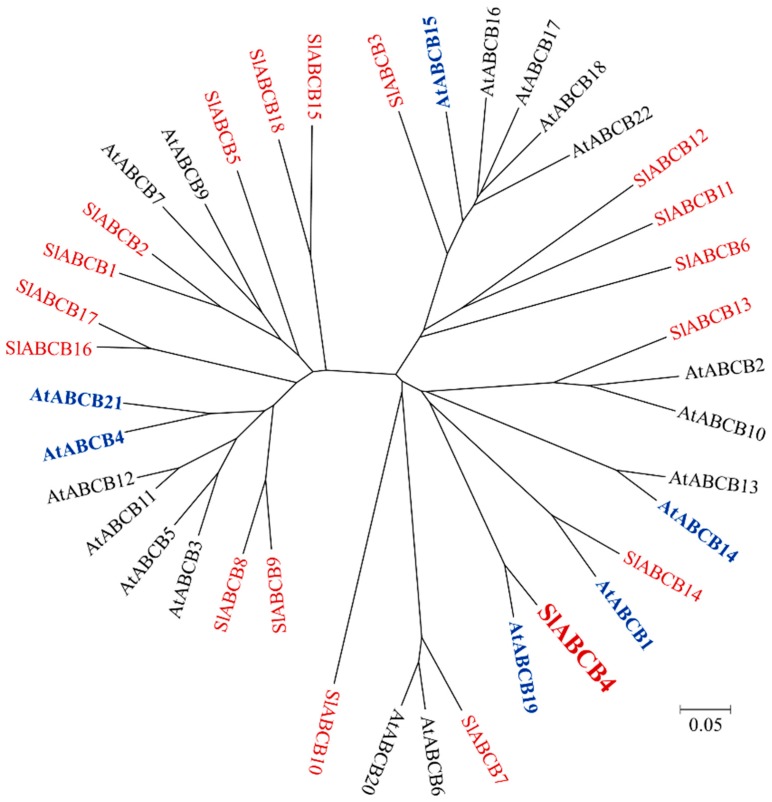
Phylogenetic tree of full-size ABCBs of tomato and Arabidopsis. Tomato ABCBs are shown in red. Arabidopsis ABCBs, characterized as auxin transporters, are indicated in blue and the others in black. The scale bar shows a 5% divergence between protein sequences [25].

In the tomato genome, the ABCB subfamily is the second largest subfamily in the ABC transporter family, which is made up of 29 members, including 18 full-size, 8 half-size and 3 quarter-size ABCBs [25]. At least six full-size ABCBs, belonging to the Arabidopsis ABCB subfamily, were characterized as auxin transporters [2] (Figure 1). These Arabidopsis ABCB auxin transporters share an evolutionary relationship with some full-size tomato ABCBs (Figure 1). For instance, tomato SlABCB4 and SlABCB14 are very similar to Arabidopsis AtABCB19 and AtABCB1, respectively. This suggests that SlABCB4 and SlABCB14 transport auxin in tomato.

We searched full-length cDNA databases of the Sol Genomics Network (https://solgenomics.net/) [35] and the TOMATOMICS (http://plantomics.mind.meiji.ac.jp/tomatomics/) [36], however, only the full length cDNA of SlABCB4 was available. Gene expression patterns of *SlABCB4* were high at the early stages of fruit development [25], hence, SlABCB4 was investigated in this study.

**Figure 2:**
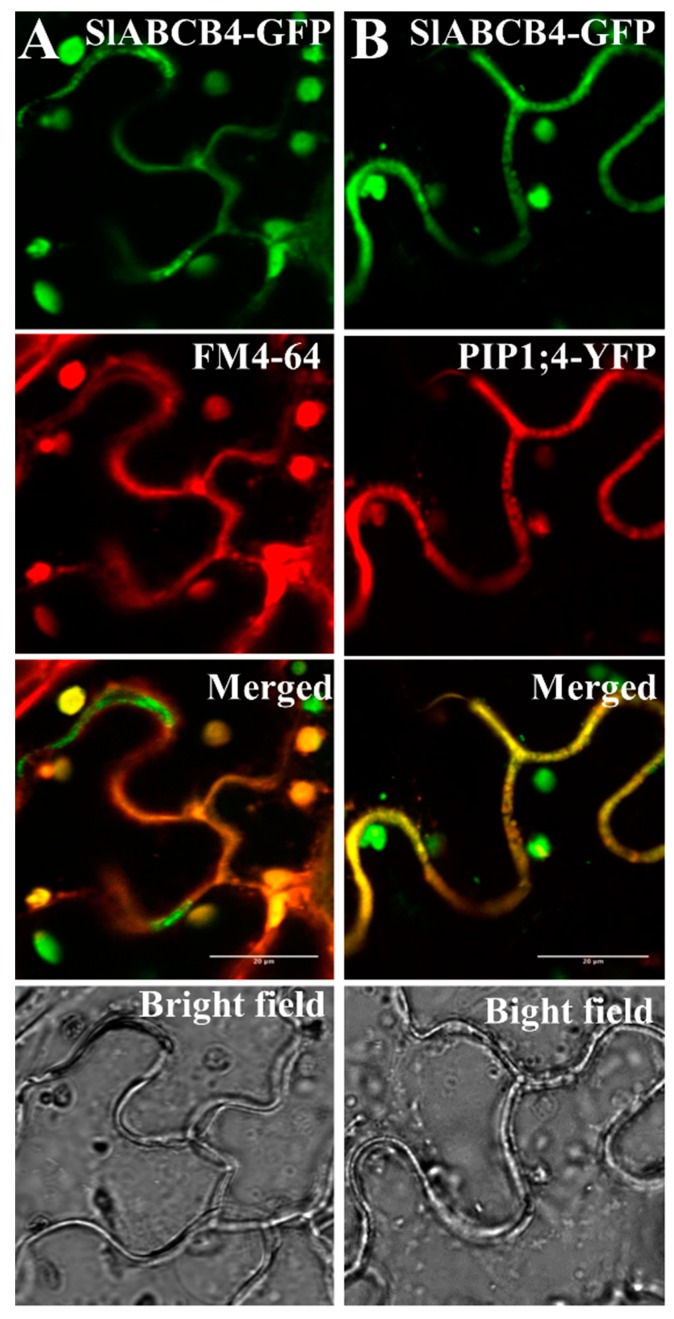
Subcellular localization of SlABCB4 in *Nicotiana benthamiana* epidermal cells. (**A**) Localization of SlABCB4-GFP fusion proteins with FM4-64 dye at the plasma membrane (PM). (**B**) Localization of SlABCB4-GFP fusion proteins and PIP1;4-mCherry fusion proteins, a PM marker. Top to down: Green fluorescence of SlABCB4-GFP fusion proteins; red fluorescence of FM4-64 dye or PIP1;4-mCherry fusion proteins, merged image of top and second pictures; and bright-field microscope images.

The subcellular localization of SlABCB4 was determined by the transient expression of the SlABCB4-GFP fusion protein in the epidermal cells of *N. benthamiana* leaves. In the epidermal cells, SlABCB4-GFP fusion proteins showed co-localization with the staining of plasma membrane by FM4-64 dye (Figure 2A). In addition, SlABCB4-GFP fusion proteins were co-localized with PIP1;4-mCherry fusion proteins (Figure 2B), which is a marker for plasma membranes [33].

**Figure 3:**
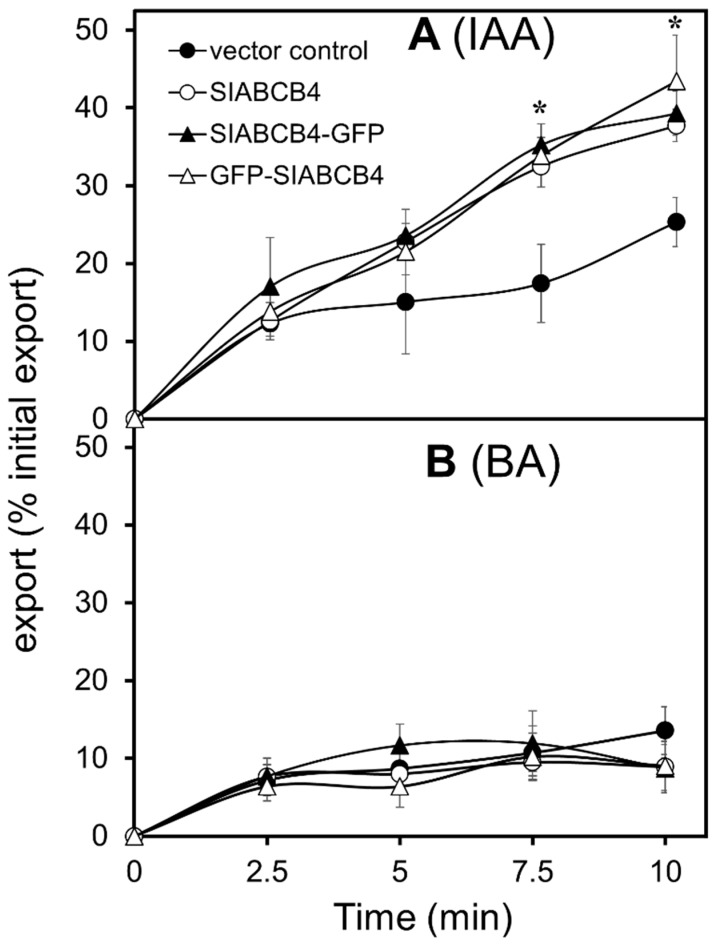
Transport assay of SlABCB4 in *Nicotiana benthamiana* protoplast. Export assay of (**A**) indole-3-acetic acid (IAA) and (**B**) benzoic acid (BA). IAA and BA transport activities in *Nicotiana benthamiana* protoplast, expressing SlABCB4, SlABCB4-GFP or GFP-SlABCB4 and vector control, were measured. Error bars show standard error for at least four experimental repeats. The asterisk is significant at *p* < 0.05.

Next, we performed a transport assay in the *N. benthamiana* protoplast expressing SlABCB4. Simultaneous IAA and BA transport assays revealed IAA export activity, but not BA export activity (Figure 3B). These results suggest a role of SlABCB4 in auxin export from cytosol to apoplast [37], as previously reported for AtABCB1, AtABCB4 and AtABCB19 [38]. 

**Figure 4:**
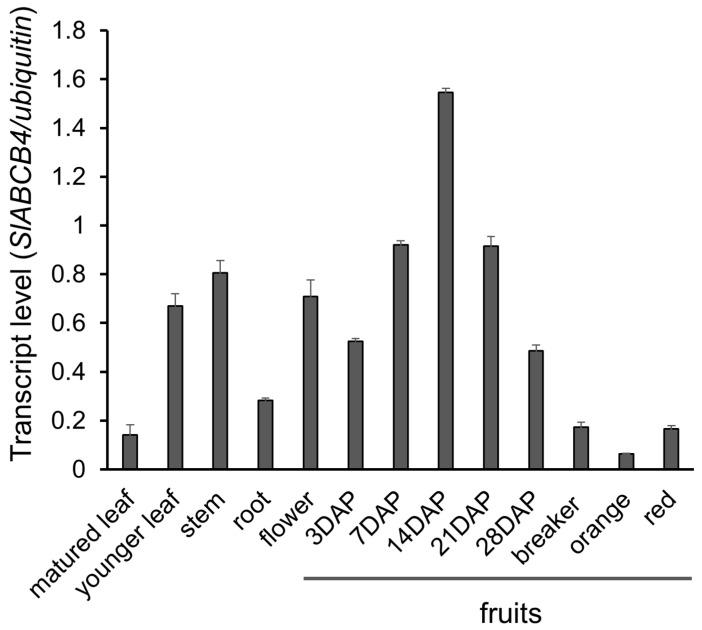
Gene expression analysis of SlABCB4 in various tomato organs. RNAs were extracted from indicated organs of ‘MicroTom’. Transcript levels of SlABCB4 were detected by RT-qPCR. The ubiquitin gene was used as a constitutively expressed control gene. Error bars are standard errors of 3 independent experiments. DAP: days after pollination.

To understand the physiological roles of SlABCB4 in tomato, gene expressions of *SlABCB4* in various organs and tissues (i.e., leaf, root, flower and developing fruits) were determined by RT-qPCR. Moderate *SlABCB4* expressions were detected in the flower, young leaf and stem (Figure 4). In the root and mature leaf, low gene expressions of *SIABCB4* were detected. The *SlABCB4* expressions were higher at early stages of fruit development, with the highest at 14 DAP (Figure 4). The gene expression level declined after 21 DAP and continued to decline at fruit maturation and ripening. These results suggest that SlABCB4 plays a more important role in the early stages of tomato fruit development than at other stages. It is proposed that the distribution of auxin at the early stage of fruit development is important for regulating cell division and expansion [14]. In tomato, cell division occurs between 7–14 DAP, followed by cell expansion, which may occur between 10–45 DAP [13,39]. Therefore, the highest expression of *SlABCB4* in 14 DAP may show an important role of SlABCB4 in auxin distribution for cell division and expansion of tomato fruit [40].

The silico gene expression profile of *SlABCB4* revealed high constitutive gene expression levels in seeds, columella, placenta, septum, pericarp and locular tissues of developing tomato fruit (http://tea.solgenomics.net/) [41]. This also shows an important role of SlABCB4 auxin transport from seeds to other fruit tissues. 

Functional characterization of *SlABCB4* RNAi knock-down tomato plants resulted in no clear phenotypic differences compared with the wild type tomato plants. This might have been caused by the existence of functional redundancy of other auxin transporters. Similarly, the silencing of SlPIN4 and SlPIN3 did not affect fruit development, but, instead, it exhibited an altered shoot architecture [14]. The Arabidopsis *atabcb1 atabcb19* plants showed reduced growth, decreased apical dominance and impaired polar auxin transport [42]. 

Two-hybrid and co-immunoprecipitation analyses indicated that AtPIN1 can interact with AtABCB1 and AtABCB19 [42], which suggests that auxin-related transporters act independently of each other. Therefore, it is most likely that SlABCB4 may coordinate with other auxin-related genes to regulate auxin distribution in tomato fruit. For fruit development, the auxin biosynthetic enzymes, TAR2 and toFZY6, and the auxin transporter, PIN5, are preferentially expressed in the seed [4]. This suggests that these auxin biosynthetic enzymes may coordinate with SlABCB4 and PIN5 to regulate auxin biosynthesis and distribution of auxin in fruit development [4]. Furthermore, SlABCB4 may coordinate with other placenta-localized auxin transporters, such as PINs (PIN1-4, PIN8) and AUX/LAX (LAX2), to transport auxin from seeds [4]. It would be very fascinating to clarify how all these auxin transporters coordinate with each other to regulate auxin distribution in the development of tomato fruit. 

## 4. Conclusions

This study presents the functional characterization of SlABCB4, a plasma membrane localized ABC transporter, and provides further evidence that SlABCB4 plays an important role in auxin transport in the development of tomato fruit. Our findings have important implications for understanding the roles of auxin-related ABC transporters in fleshy fruit development.

## Supplementary Materials

The following are available online at http://www.mdpi.com/2223-7747/7/3/65/s1, Figure S1: Tissue expression analysis of SlABCB4 in developing fruit. High-resolution transcript data of SlABCB4 was obtained from Tomato Expression Atlas (Tea; http://tea.solgenomics.net/).
