# Supplementary material for: Tomato ATP-Binding Cassette Transporter SlABCB4 Is Involved in Auxin Transport in the Developing Fruit
Source: Plants (Basel). 2018 Aug 13;7(3):65. doi: 10.3390/plants7030065 (PMC6161087; doi:10.3390/plants7030065)
Supplement: Supplementary file 1 [file plants-07-00065-s001.zip › Figure S1.pptx]

## Slide 1
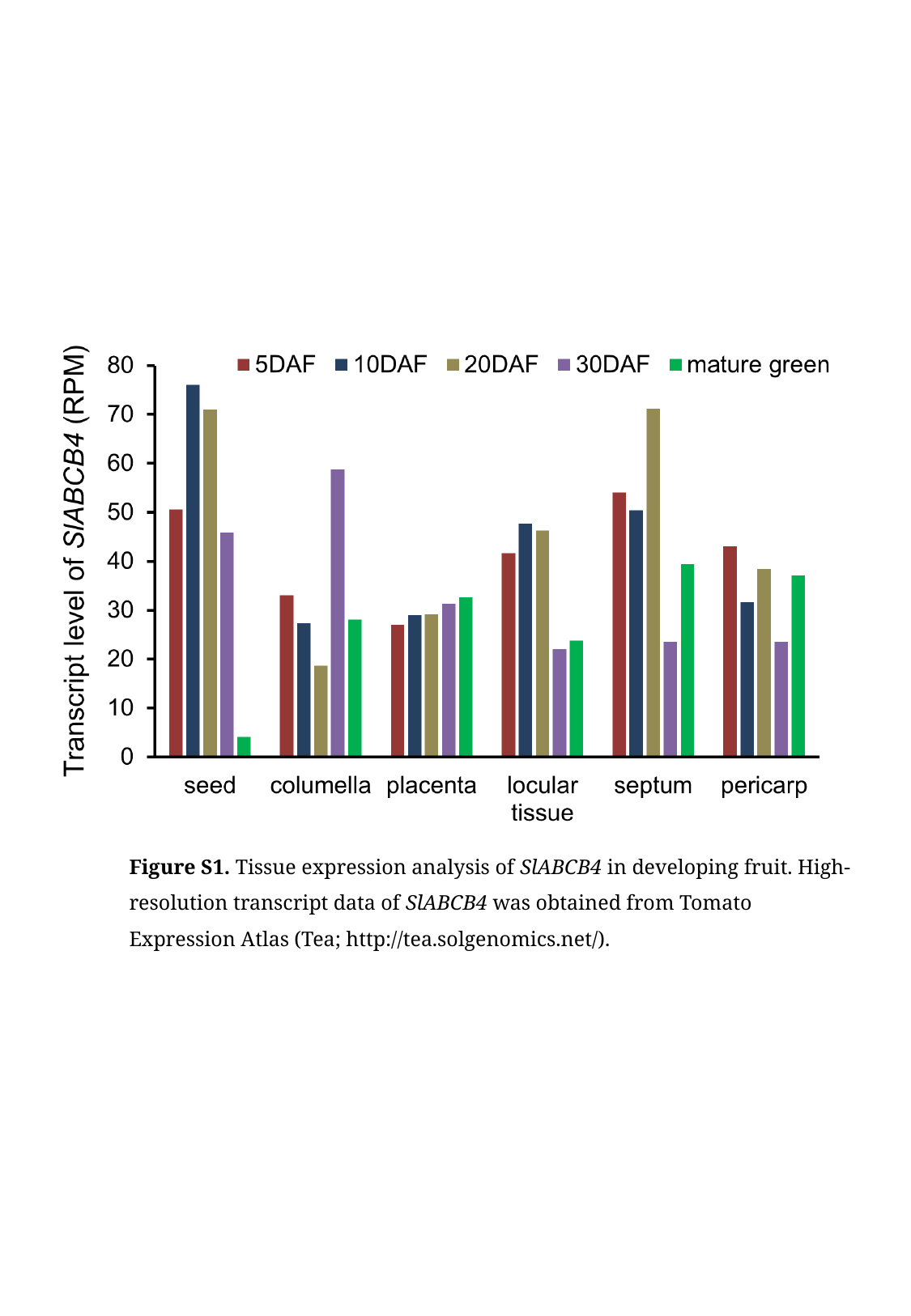

Figure S1. Tissue expression analysis of SlABCB4 in developing fruit. High-resolution transcript data of SlABCB4 was obtained from Tomato Expression Atlas (Tea; http://tea.solgenomics.net/).
