# Supplementary material for: Tomato ATP-Binding Cassette Transporter SlABCB4 Is Involved in Auxin Transport in the Developing Fruit
Source: Plants (Basel). 2018 Aug 13;7(3):65. doi: 10.3390/plants7030065 (PMC6161087; doi:10.3390/plants7030065)
Supplement: Supplementary file 1 [file plants-07-00065-s001.zip › Table S1.docx]

**Supplementary Table 1: Primers designed and used in this study**

| Primers for reverse transcription-quantitative (RT-qPCR) | |
| --- | --- |
| Forward | 5’- GCAATCAACATGGCCTAGTG -3’ |
| Reverse | 5’- AAAGTCACTAGGACCCAAACA -3’ |
| *Ubiquitin* | |
| Forward | 5’- CACCAAGCCAAAGAAGATCA -3’ |
| Reverse | 5’- TCAGCATTAGGGCACTCCTT -3’ |
| PCR condition | 98℃ 10s, 55℃ 30s, 72℃ 10s (40 cycles) |
| Infusion cloning primers for pENTR/D-TOPO vector and SlABCB4 | |
| Forward | 5’- GCCGCCCCCTTCACCATGGCGGAGACTACTGAAGGG-3’ |
| Reverse | 5’- GGCGCGCCCACCCTTTATGCGGTGGTGTTGTAGTTGC-3’ |
| Primers for linearization of pENTR/D-TOPO vector | |
| Forward | 5’-AAGGGTGGGCGCGCCGAC-3’ |
| Reverse | 5’-GGTGAAGGGGGCGGCCGC-3’ |
